# Causes of Admission, Prescribing Patterns, and Outcomes Among Trauma Patients Visiting the Department of Trauma and Emergency at a Tertiary Care Hospital in Bihar, India: A Cross-Sectional Study

**DOI:** 10.7759/cureus.115821

**Published:** 2026-09-05

**Authors:** Kumari Pallavi, Keshav Kumar, Anshuman Chandra, Kumar Martand, Lalit Mohan, Manish Kumar, Prem Prakash

**Affiliations:** 1 Pharmacology and Therapeutics, Indira Gandhi Institute of Medical Sciences, Patna, IND; 2 Pharmacology and Therapeutics, Hi-Tech Medical College and Hospital, Bhubaneswar, IND; 3 Anatomy, Employees' State Insurance Corporation (ESIC) Medical College, Kolkata, IND; 4 General Surgery, Indira Gandhi Institute of Medical Sciences, Patna, IND

**Keywords:** burden, injury, outcome, prescribing pattern, prevalence, trauma

## Abstract

Introduction

Trauma is one of the most common, preventable causes of mortality globally. Its burden is rising, particularly in low- and middle-income nations, and hence, it is being acknowledged as an epidemic. The factors responsible for the high burden of trauma and trauma-related morbidity and mortality in resource-limited countries need to be identified and quantified. Additionally, there is a paucity of data from our setting on prescribing patterns among trauma victims. We, therefore, aimed to evaluate the burden, attributes, and outcomes of trauma as well as the prescribing patterns among trauma victims.

Objectives

The primary objective was to evaluate the burden, characteristics, and outcomes of trauma, whereas the secondary objective was to evaluate the prescribing patterns among trauma patients visiting the Department of Trauma and Emergency.

Methods

This was a hospital record-based, cross-sectional study conducted in the Department of Trauma and Emergency in collaboration with the Department of Pharmacology at Indira Gandhi Institute of Medical Sciences (IGIMS), Patna, which required a sample size of 369. The primary outcome was the prevalence of trauma, its characteristics, and death, presented as numbers with percentages, whereas the secondary outcome was the frequency of drugs prescribed from various classes.

Results

Out of 4,642 patients who visited the trauma and emergency department during the study, 1,137 were trauma patients. Thus, the prevalence of trauma in the study population was 24.49%. People aged between 20 and 40 years were the most affected (193, 52.30%), and most of the trauma victims were men (293, 79.40%). Rural people (284, 76.96%) were about four times more affected than urban ones (85, 23.04%). The street/highway was the most common place (124, 33.60%) for incidents, the time between 16:00 and 19:59 hours was the most common time of occurrence of incidents (93, 25.20%), and the ambulance was the most common mode of transportation (148, 40.11%) of trauma victims. Road traffic accidents were the most common cause of trauma, accounting for 152 (41.19%) of cases, while head injuries were the most common type of injury reported in 132 (35.77%) of cases. Analgesics, antibiotics, antiulcer and antiemetics, and intravenous fluids were major classes of drugs prescribed in 369 (100%), 369 (100%), 323 (87.53%), and 281 (76.19%) of cases, respectively. Out of 369 trauma patients under study, 28 (7.59%) cases died.

Conclusion

The prevalence of trauma among patients admitted to the trauma and emergency department was 20%. Road traffic accident was the most common cause of trauma, while head injury was the most common type of injury. Among the various classes of drugs, analgesics and antibiotics were the most consistently prescribed drugs. About 8% of trauma patients admitted to the trauma and emergency department died.

## Introduction

The World Health Organization (WHO) defines injury as the damage caused by physical, thermal, chemical, and radiation energies that exceeds the physiological threshold of the body [1]. Trauma is the third most prevalent cause of death globally and is a major cause of morbidity and mortality, especially for people under 40 years of age [2,3]. There is a rising trend in all forms of injuries globally, resulting in grave consequences and disability, and about 9% of deaths are attributable to them. Every day, about 1.6 thousand people die due to injuries across the world [4,5]. In India, 19 people die every hour due to road traffic injuries, which translates to 9.5 deaths for every 100,000 people [6,7]. Trauma is being acknowledged as a current epidemic, particularly in low- and middle-income nations, and to a great extent, it is preventable [8,9]. Given the rapid urbanization, industrialization, and motorization coupled with strained and overcrowded road traffic systems and limited healthcare budgets, trauma has become a silent killer [10].

There are considerable variations in the proportions of trauma and trauma-related morbidity and mortality among different nations, with low- and middle-income countries having higher proportions [11-14]. Patients visiting the trauma and emergency (T&E) department encounter many challenges, including overcrowding, inadequate communication, lack of empathy, uncomfortable environments, and a lack of well-trained healthcare providers [15-17]. Emergency situations may also lead to irrational prescribing, mostly in the form of polypharmacy and use of reserve antibiotics. By knowing the cause of injury, preventive measures through public awareness and policymaking can be taken and strengthened. By knowing the fate of trauma victims, healthcare providers and policymakers can plan strategies to decrease mortality and improve outcomes. Knowledge of prescribing patterns helps to curtail irrational prescribing, including the use of reserve antibiotics, which in turn aids in improving overall patient outcomes. With this background, we aimed to evaluate the burden, characteristics, and outcomes of trauma patients along with prescribing patterns.

Objectives

The primary objective was to evaluate the burden, characteristics, and outcomes of trauma among patients visiting the T&E department, whereas the secondary objective was to evaluate the prescribing patterns among trauma victims admitted in the T&E department.

## Materials and methods

Study design, setting, and duration

This was a hospital record-based cross-sectional study conducted in the Department of Trauma and Emergency in collaboration with the Department of Pharmacology at Indira Gandhi Institute of Medical Sciences (IGIMS), Patna. IGIMS has about 1,800 beds allocated to different specialties and super-specialties, including about 85 fully equipped beds dedicated to the T&E department. Being a tertiary care hospital, IGIMS caters to local and regional patients from the same and neighboring states. The total duration of the study was six months, from April 21, 2025, to October 20, 2025.

Inclusion and exclusion criteria

Patients of any age who sustained trauma of any type and were admitted to the T&E department were included in the study. Patients who were not candidates for admission in the T&E department, were referred to other centers after admission, left the hospital against medical advice, or had inadequate information on their prescriptions were excluded from the study.

Sample size calculation

The calculation of required sample size was based on the prevalence of injury in a previous study [1] and was performed for a single proportion using the online calculator Statulator.com. Assuming the same prevalence of trauma as in the previous study, i.e., 57%, and two-tailed hypotheses, the study required a sample size of 369 for estimating the expected proportion with 5% absolute precision and 95% confidence.

Sampling technique

The source population was the patients visiting the Department of Trauma and Emergency, and the study population was the trauma patients visiting the Department of Trauma and Emergency during the study period. Consecutive sampling was used to achieve the sample size. Records of all patients visiting the Department of Trauma and Emergency were identified from the departmental patients’ logbook.

Outcome

The primary outcome was the proportion of trauma, its characteristics, and death of trauma patients, presented as numbers with percentages, while the secondary outcome was the frequency of drugs prescribed from various classes, presented as numbers with percentages.

Data collection procedure

A pretested and structured data extraction form adapted from WHO injury surveillance guidelines and validated for low- and middle-income countries was used to collect the data (Appendix). The data collector was given training on the content of the extraction form and how to collect the data. To maintain data quality, the supervisor re-checked the collected data daily and submitted the same to the principal investigator. The extraction form consisted of socio-demographic characteristics (age, sex, place of residence, and education level), clinical characteristics (systolic blood pressure, diastolic blood pressure, pulse rate, and respiratory rate), trauma-related parameters (place of occurrence, time of incidents, modes of transportation, time to arrive at the T&E department, mechanism of trauma, type of injury, and duration of hospital stay), various classes of drug/products prescribed, and outcomes of trauma victims.

Statistical analysis

After checking for completeness and consistency, data were entered into a Microsoft Excel sheet (Microsoft Corporation, Redmond, WA, USA). Descriptive analytics were used to present data. All data were analyzed using software Jamovi (version 2.6.26; The jamovi project, Sydney, Australia).

Ethical consideration

The study was initiated after obtaining approval from the Institutional Ethics Committee (Ref no. 362/IEC/IGIMS/2025; dated March 17, 2025) and was conducted following good clinical practice and in accordance with the principles of the Declaration of Helsinki.

## Results

Consecutive patients visiting the T&E department were screened for eligibility. In order to achieve the sample size, we screened records of 4,642 patients who visited the T&E department during the study, out of which 1,137 were trauma patients. Thus, the prevalence of trauma in the source population was 24.49%. Out of the remaining 1,137 patients, 205 left the hospital against medical advice, 563 had incomplete records, and 369 were included in the study (Figure 1).

**Figure 1:**
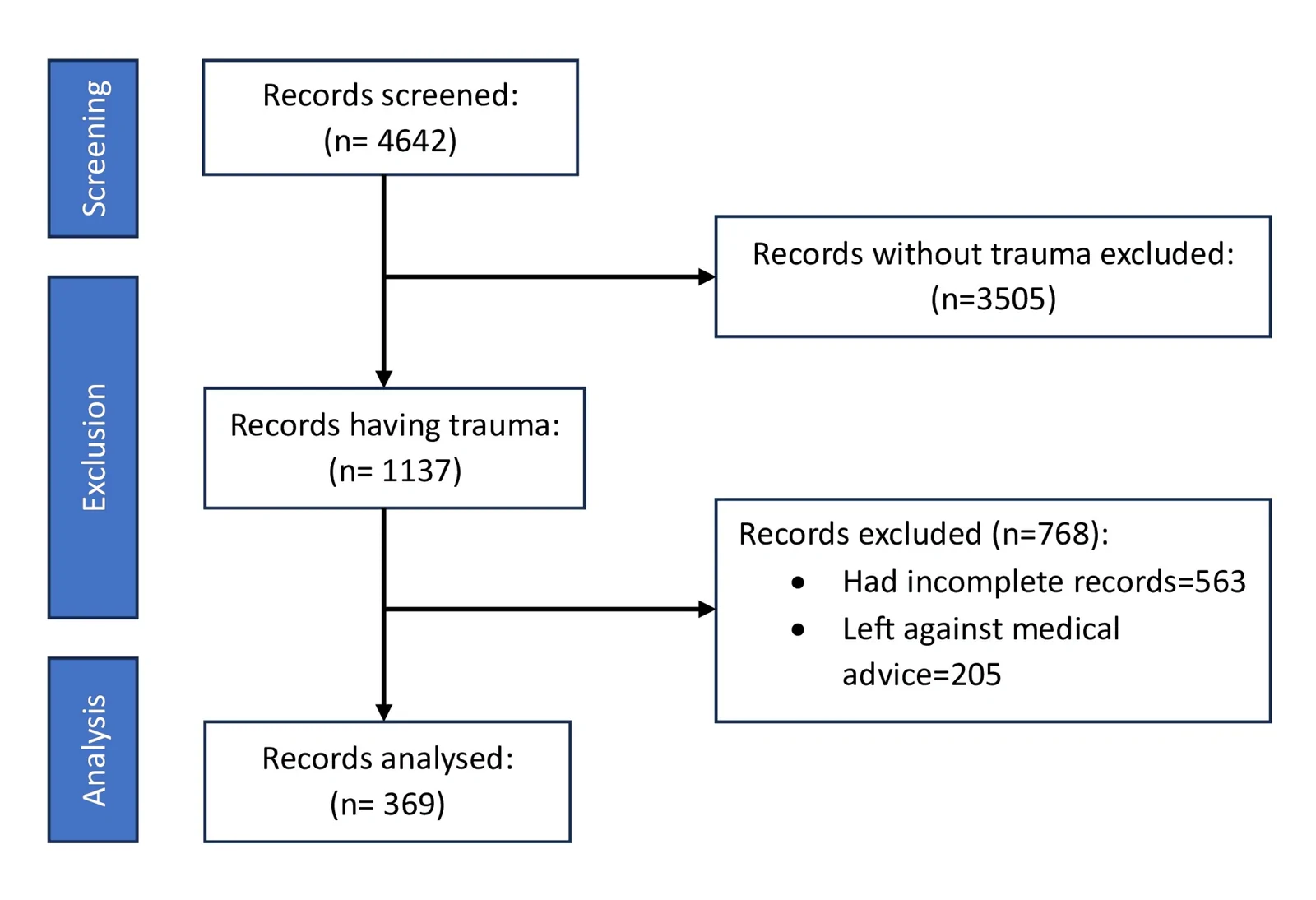
Flow diagram showing screening, inclusion, exclusion, and analysis of patients' records

Socio-demographic characteristics

The mean age of trauma victims was 26.65 (±14.32) years. About 79.40% (293) of trauma patients were male, 19.24% (71) were female, and 1.36% (5) were third gender, with a high male-to-female ratio of 4.13:1. About 76.96% (284) of trauma victims were rural residents, and the remaining 23.04% (85) were urban dwellers. About 19.24% (71) trauma victims were not able to read and write, 25.47% (94) had primary, 30.35% (112) had secondary, 15.45% (57) had graduate, 8.67% (32) had post-graduate, and 0.81% (3) had post-doctoral levels of education (Table 1).

**Table 1:** Socio-demographic characteristics of trauma patients

Characteristics	Category	Frequency, n (%)	Outcome	
			Did not die, n (%)	Died, n (%)
Age (years)	<20	77 (20.87)	72 (93.51)	5 (6.49)
	20-40	193 (52.30)	178 (92.22)	15 (7.78)
	40-60	54 (14.63)	50 (92.59)	4 (7.41)
	>60	45 (12.19)	41 (91.11)	4 (8.89)
Sex	Male	293 (79.40)	270 (92.15)	23 (7.85)
	Female	71 (19.24)	66 (92.96)	5 (7.04)
	Third gender	5 (1.36)	5 (100)	0 (0)
Residence	Rural	284 (76.96)	262 (92.25)	22 (7.75)
	Urban	85 (23.04)	79 (92.94)	6 (7.06)
Education level	Illiterate	71 (19.24)	65 (91.55)	6 (8.45)
	Primary	94 (25.47)	86 (91.49)	8 (8.51)
	Secondary	112 (30.35)	103 (91.97)	9 (8.03)
	Graduate	57 (15.45)	54 (94.74)	3 (5.26)
	Post-graduate	32 (8.67)	30 (93.75)	2 (6.25)
	Post-doctoral	3 (0.81)	3 (100)	0 (0)

Vital clinical parameters of trauma victims

The Glasgow Coma Scale (GCS) score of 298 (80.76%) of trauma victims was between 13 and 15, that of 26 (7.05%) was between 9 and 12, and that of the remaining 45 (12.19%) was ≤8. Based on systolic blood pressure, 43 (11.65%) trauma patients were found to be hypotensive, whereas 326 (88.35%) were normotensive. Pulse rates of 271 (73.44%) of trauma victims were in the normal range (60-100 bpm), those of 88 (23.85%) were in the tachycardic range (>100 bpm), and those of 10 (2.71%) were in the bradycardic range (<60 bpm) (Table 2).

**Table 2:** Vital clinical characteristics of trauma victims GCS: Glasgow Coma Scale; SBP: systolic blood pressure

Characteristics	Category	Frequency, n (%)	Outcome	
			Did not die, n (%)	Died, n (%)
GCS score	13-15	298 (80.76)	283 (94.97)	15 (5.03)
	9-12	26 (7.05)	23 (88.46)	3 (11.54)
	≤8	45 (12.19)	35 (77.78)	10 (22.22)
SBP	Hypotensive	43 (11.65)	37 (86.05)	6 (13.95)
	Normotensive	326 (88.35)	304 (93.25)	22 (6.75)
Pulse rate	Bradycardia (<60 bpm)	10 (2.71)	8 (80.00)	2 (20.00)
	Normal (60-100 bpm)	271 (73.44)	254 (93.73)	17 (6.27)
	Tachycardia (>100 bpm)	88 (23.85)	79 (89.77)	9 (10.23)

Trauma characteristics

Frequency of key trauma-related parameters such as place of occurrence, time of occurrence, mode of transportation, time to arrive at the T&E department, mechanism of trauma, type of injury, and duration of hospital stay along with outcomes of trauma victims are presented in Table 3 and Figure 2.

**Table 3:** Trauma characteristics and other related parameters RTA: road traffic accidents

Parameters	Categories	Number (%)	Outcome	
			Did not die, n (%)	Died, n (%)
Place of occurrence	Street/highway	124 (33.60)	111 (89.52)	13 (10.48)
	Home	45 (12.19)	43 (95.56)	2 (4.44)
	Sports and athletics area	38 (10.30)	35 (92.11)	3 (7.89)
	Industrial/construction area	63 (17.07)	57 (90.48)	6 (9.52)
	School	12 (3.25)	11 (91.67)	1 (8.33)
	Farm	47 (12.74)	46 (97.79)	1 (2.21)
	Others	40 (10.84)	38 (95.00)	2 (5.00)
Time of occurrence	00:00-03:59 hours	22 (5.69)	19 (86.36)	3 (13.64)
	04:00-07:59 hours	64 (17.34)	60 (93.75)	4 (6.25)
	08:00-11:59 hours	77 (20.87)	71 (92.21)	6 (7.79)
	12:00-15:59 hours	31 (8.40)	28 (90.32)	3 (9.68)
	16:00-19:59 hours	93 (25.20)	85 (91.40)	8 (8.60)
	20:00-23:59 hours	34 (9.21)	32 (94.12)	2 (5.88)
	Unknown	48 (13.01)	46 (95.83)	2 (4.17)
Mode of transportation	Ambulance	148 (40.11)	140 (94.60)	8 (5.40)
	Autorickshaw/electric rickshaw	93 (25.20)	86 (92.47)	7 (7.53)
	Cabs/taxi	62 (16.80)	56 (90.32)	6 (9.68)
	Two wheelers	41 (11.11)	37 (90.24)	4 (9.76)
	Police van	25 (6.77)	22 (88.00)	3 (12.00)
Time to arrive at the trauma and emergency department	Immediate (<1 hour)	34 (9.21)	33 (97.06)	1 (2.94)
	Within hours (1-24 hours)	186 (50.41)	174 (93.55)	12 (6.45)
	Within days (>24 hours)	149 (40.38)	134 (89.93)	15 (10.07)
Mechanism of trauma	RTA	152 (41.19)	138 (90.79)	14 (9.21)
	Assaults/interpersonal violence	114 (30.89)	107 (93.86)	7 (6.14)
	Falls	55 (14.90)	52 (94.55)	3 (5.45)
	Burns	26 (7.05)	24 (92.31)	2 (7.69)
	Choking	13 (3.52)	12 (92.31)	1 (7.69)
	Others	9 (2.44)	8 (92.89)	1 (11.11)
Type of injury	Head	132 (35.77)	119 (90.15)	13 (9.85)
	Polytrauma	95 (25.74)	87 (91.58)	8 (8.42)
	Upper trunk	23 (6.23)	21 (91.31)	2 (8.69)
	Abdomen and pelvis	42 (11.38)	40 (95.24)	2 (4.76)
	Upper limb	21 (5.69)	20 (95.24)	1 (4.76)
	Lower limb	56 (15.18)	54 (96.43)	2 (3.57)
Duration of hospital stay	<7 days	221 (59.89)	211 (95.48)	10 (4.52)
	≥7 days	148 (40.11)	130 (87.84)	18 (12.16)

**Figure 2:**
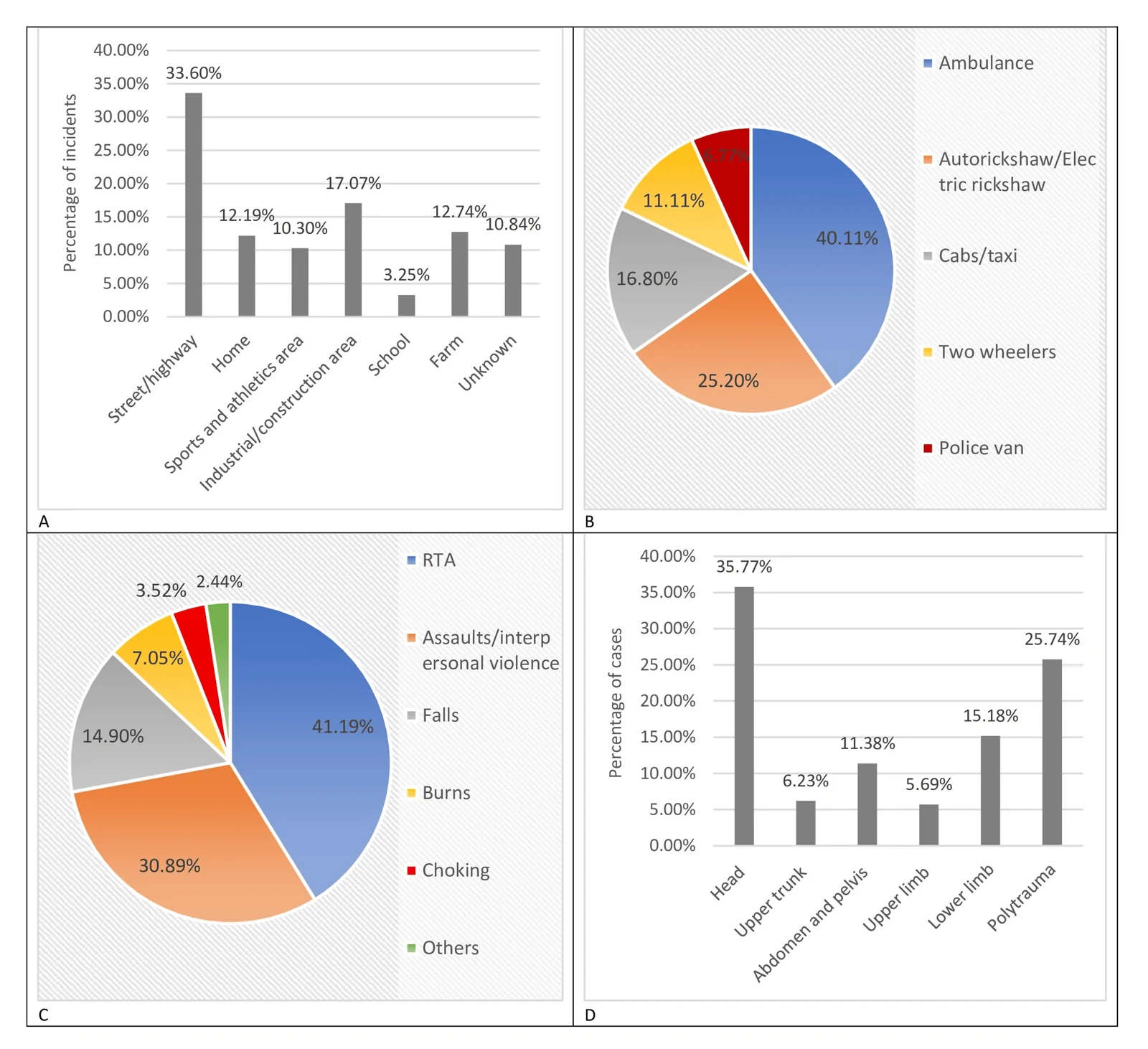
Characteristics of trauma (A) Place of occurrence of incidents; (B) mode of transportation of trauma victims; (C) mechanism of trauma; (D) type of traumatic injuries RTA: road traffic accidents

Street/highway was the most common place of incident occurrence involving 124 (33.60) followed by industrial/construction area, farm, home, unknown places, sports/athletics area, and school, respectively, involving 63 (17.07%), 47 (12.74%), 45 (12.19%), 40 (10.84%), 38 (10.30%), and 12 (3.25%) of incidents (Table 3, Figure 2A). Times from 16:00 to 19:59 hours and from 08:00 to 11:59 hours were the two peak hours, observing 93 (25.20%) and 77 (20.87%) of incidents, respectively (Table 2).

Ambulance was the most common mode of transportation, which brought 148 (40.11%) of trauma victims, followed by autorickshaw/electric rickshaw, cabs/taxi, two wheelers, and police van, which brought 93 (25.20%), 62 (16.80%), 41 (11.11%), and 25 (6.77%) of trauma victims to the T&E department, respectively (Table 3, Figure 2B). One hundred eighty-six (50.41%) of trauma victims arrived at the T&E department between 1 and 24 hours after the incident, 149 (40.38%) after 24 hours, and 34 (9.21%) within one hour of the incident (Table 3). Road traffic accident (RTA) was the most common mechanism of trauma accounting for 152 (41.19%) followed by assaults/interpersonal violence, falls, burns, choking, and other mechanisms accounting for 114 (30.89%), 55 (14.90%), 26 (7.05%), 13 (3.52%), and 9 (2.44%) of incidents, respectively (Table 3, Figure 2C).

Head injury was the most common type of injury seen in 132 (35.77%) followed by polytrauma, lower limb injury, abdomen and pelvis injury, upper trunk injury, and upper limb injury, respectively, seen in 95 (25.74%), 56 (15.18%), 42 (11.38%), 23 (6.23%), and 21 (5.69%) of trauma patients (Table 3, Figure 2D). One hundred forty-eight (40.11%) of trauma victims had a duration of hospital stay of ≥7 days, whereas 221 (59.89%) had a duration of hospital stay of <7 days.

Prescribing pattern

Various classes of drugs/products prescribed to trauma patients were analgesics, intravenous fluids, antiulcer and antiemetic drugs, blood and blood products, sedative/emergency airway drugs, vasopressors, anticoagulants, antibiotics, antitetanus toxoid/immunoglobulins, and some miscellaneous drugs (Table 4).

**Table 4:** Classes and frequency of drugs/products prescribed to trauma victims FDC: fixed-dose combination

Classes of drugs/products	Number (percentage)	Indications
1. Analgesics		
A. Opioids		Management of acute trauma pain
Tramadol	162 (43.90)	
Morphine	74 (20.05)	
Fentanyl	68 (18.43)	
B. Non-opioids		
Diclofenac	267 (72.36)	
Aceclofenac/paracetamol FDC	282 (76.42)	
Paracetamol	136 (36.86)	
2. Intravenous fluids		
Normal saline	281 (76.15)	Fluid resuscitation, shock management
Ringer’s lactate	42 (11.38)	
Mannitol	173 (46.88)	Prevention of cerebral edema
3. Antiulcer and antiemetics		
Pantoprazole	251 (68.02)	Stress ulcer prophylaxis
Omeprazole	38 (10.29)	
Ondansetron	183 (49.59)	Prevention and control of nausea and vomiting
4. Blood and blood products		
Packed red blood cells (PRBCs)	97 (26.29)	Acute hemorrhage, hemorrhagic shock, anemia due to blood loss
Fresh frozen plasma (FFP)	43 (11.65)	Trauma-induced coagulopathy, massive transfusion protocols
Platelet concentrates	35 (9.48)	Thrombocytopenia, massive bleeding, coagulopathy
Cryoprecipitate	11 (2.98)	Hypofibrinogenemia during severe hemorrhage
Whole blood	7 (1.89)	Massive hemorrhage
5. Sedative/emergency airway drugs		
Midazolam	95 (25.74)	Procedures or emergency airway
Ketamine	114 (30.89)	
6. Vasopressors		
Noradrenaline	57 (15.45)	To treat hypotension and cardiogenic shock
Vasopressin	12 (3.25)	
Adrenaline	5 (1.35)	
7. Anticoagulants		
Enoxaparin	234 (63.41)	Prevention of deep vein thrombosis
Rivaroxaban	116 (31.44)	
Fondaparinux	32 (8.67)	
8. Antibiotics		
Ceftriaxone	221 (59.89)	Treatment of wound or surgical site infections
Amoxicillin-clavulanate	138 (37.40)	
Cefuroxime	84 (22.76)	
Cefotaxime	92 (24.93)	
Cefixime	66 (17.87)	
Azithromycin	48 (13.01)	
Ciprofloxacin	35 (9.48)	
Amikacin	74 (20.05)	
Gentamicin	52 (14.09)	
Metronidazole	104 (28.18)	
Clindamycin	96 (26.02)	
Meropenem	32 (8.67)	
Piperacillin-tazobactam	28 (7.59)	
9. Antitetanus prophylaxis		
Tetanus toxoid	193 (52.30)	Tetanus prophylaxis
Tetanus immunoglobulin	140 (37.94)	
10. Others		
Tranexamic acid	104 (28.18)	Control of acute bleeding
Phenytoin	47 (12.74)	Control of seizures in traumatic brain injury
Levetiracetam	84 (22.76)	

In many patients, multiple drugs were prescribed from the same classes, injectables followed by oral tablets/capsule. Among analgesic drugs, tramadol, morphine, and fentanyl were the opioid analgesics prescribed in, respectively, 162 (43.90%), 74 (20.05%), and 68 (18.43%), whereas diclofenac, aceclofenac/paracetamol fixed-dose combination (FDC), and paracetamol were non-opioids prescribed in, respectively, 267 (72.36%), 282 (76.42%), and 136 (36.86%) of the trauma victims. Among intravenous fluids, normal saline and Ringer’s lactate were the crystalloids prescribed in 281 (76.15%) and 42 (11.38%), respectively, whereas mannitol was the colloid prescribed in 173 (46.88%) of trauma victims. Pantoprazole and omeprazole were the antiulcer drugs prescribed in 251 (68.02%) and 38 (10.29%), respectively, whereas ondansetron was the antiemetic prescribed in 183 (49.59%) of trauma victims. Packed red blood cells (PRBCs), fresh frozen plasma (FFP), platelet concentrates, and cryoprecipitate were the blood products prescribed in 97 (26.29%), 43 (11.65%), 35 (9.48%), and 11 (2.98%), respectively, whereas whole blood was prescribed in seven (1.89%) of trauma victims. Midazolam and ketamine were the sedative/emergency airway drugs prescribed in 95 (25.74%) and 114 (30.89%) of the trauma victims, respectively. Noradrenaline, vasopressin, and adrenaline were the vasopressors prescribed in 57 (15.45%), 12 (3.25%), and 5 (1.35%) of cases, respectively, whereas enoxaparin, rivaroxaban, and fondaparinux were the anticoagulants prescribed in 234 (63.41%), 116 (31.44%), and 32 (8.67%) of cases, respectively.

At least one antibiotic was prescribed to each trauma victim. β-Lactams were the most commonly prescribed antibiotics and included ceftriaxone (221, 59.89%), amoxicillin-clavulanate (138, 37.40%), cefotaxime (92, 24.93%), cefuroxime (84, 22.76%), cefixime (66, 17.87%), meropenem (32, 8.67%), and piperacillin-tazobactam (28, 7.59%). The next common antibiotics were aminoglycosides, which included amikacin (74, 20.05%), gentamicin (52, 14.09%), metronidazole (104, 28.18%), clindamycin (96, 26.02%), azithromycin (48, 13.01%), and ciprofloxacin (35, 9.48%).

## Discussion

In this study, we found that 24.49% (1,137) of patients visiting the T&E department were trauma victims. This prevalence rate is slightly higher than that in the study from eastern India conducted by Jain et al. (19.8%) [18] but very close to that in the study from Nigeria conducted by Onyemaechi et al. (24.3%) [19].

The mean age of trauma victims was 26.65 (±14.32) years. Persons aged between 20 and 40 years were the most commonly affected, involved in 193 (52.30%) incidents, followed by teenagers and pediatric age groups collectively involved in 77 (20.87%) of incidents. This was the consistent finding across the studies by Menon et al. [20] and Jain et al. [18], wherein the most common age group of trauma victims was 20-29 and 21-30 years, respectively. The casualty rate was slightly higher in the age groups of 20-40 years (7.78%) and >60 years (8.89%), probably due to severe forms of injury in the former group and elderly victims in the latter group. Men were about four times more affected than women. This finding is consistent with WHO injury and violence surveillance facts [21] and also with studies from India conducted by Menon et al. (3.34: 1) [20], Jain et al. (4.38:1) [18], and Singh and Mishra (3.61:1) [22], and from Ethiopia conducted by Zegeye et al. (4.03:1) [1]. The high burden of trauma among young men may be justified by the fact that in India, young men are more commonly engaged in activities like driving, industrial/construction work, sports/athletic activities, and farming, which are the major activities leading to trauma. Casualties had a male preponderance, with 23 (7.85%) males and 5 (7.04%) females, respectively. In both rural and urban victims, RTA was the most common mechanism of trauma, and head injury was the most common type of injury. The next common mechanisms of trauma in rural victims were assaults/interpersonal violence, falls, burns, and choking, whereas in urban victims, they were falls, burns, assaults/interpersonal violence, and choking in decreasing frequency. The casualty rate in rural victims (7.75%) was slightly higher than in urban victims (7.06%), which might probably be due to delays for rural victims in reaching an equipped trauma center.

The GCS score of 298 (80.76%) of trauma victims was between 13 and 15, that of 26 (7.05%) was between 9 and 12, and that of the remaining 45 (12.19%) was ≤8. The mortality rates among these GCS categories were 5.03%, 11.54%, and 22.22%, respectively. Based on systolic blood pressure, 326 (88.35%) trauma patients were found to be normotensive, whereas 43 (11.65%) were hypotensive and were associated with 6.75% and 13.95% mortality, respectively. Pulse rates of 271 (73.44%) of trauma victims were in the normal range (60-100 bpm), those of 88 (23.85%) were in the tachycardic range (>100 bpm), and those of 10 (2.71%) were in the bradycardic range (<60 bpm) and were associated with 6.27%, 10.23%, and 20.00% of casualties, respectively.

Street/highway (124 (33.60%)) was the most common place of incidents, followed by industrial/construction area (63 (17.07%)), accounting for 10.48% and 9.52% of casualties, respectively. Most of the incidents of trauma occurred during 16:00-19:59 hours (93, 25.20%), 08:00-11:59 hours (77, 20.87%), and 04:00-07:59 hours (64, 17.34%) and were associated with deaths of 8.60%, 7.79%, and 13.64% of victims, respectively. Our findings align with Jain et al. [18], who reported 2-6 PM as the most common time of occurrence of trauma, and with Kashid et al. [23], who noted a high mortality rate when accidents took place during early morning. These findings may be explained by the fact that the number of trauma incidents in our study is mainly contributed by RTAs, and the above-mentioned hours are the peak hours for RTAs attributable to high traffic congestion during office/duty hours and sleepiness of vehicle drivers in the early morning.

Ambulance was the most common mode of transportation used by 148 (40.11%) trauma victims and was associated with the lowest casualty rate (5.40%) when compared with casualty rates of other modes of transportation such as autorickshaw/electric rickshaw (7.53%), cabs/taxi (9.68%), two-wheelers (9.76%), and police control room (PCR) van (12.00%). Menon et al. [20] from South India found private vehicles or taxis as the major mode of transportation (36.5%), whereas Jain et al. [18] from eastern India and Zegeye et al. [1] from Ethiopia found ambulances were the most common mode of transportation (in 57% and 35.49% of cases, respectively).

Only 34 (9.21%) of trauma victims arrived at hospitals within one hour of the incident, 186 (50.41%) arrived between 1 and 24 hours, and 149 (40.38%) arrived only after 24 hours. Only local victims were able to reach the hospital immediately. Victims from remote areas of the state or from neighboring states arrived at the hospital after a long delay because many trauma patients were first admitted to a local hospital or the travel time was long. The mortality rate was 4.17% among victims who arrived within one hour, as compared to 6.45% in those who arrived between 1 and 24 hours and 10.07% in those who arrived after 24 hours of the incident.

RTA (152, 41.19%) was the most common mechanism of trauma, followed by assaults/interpersonal violence (114, 30.89%), falls (55, 14.90%), burns (26, 7.05%), choking (13, 3.52%), and miscellaneous mechanisms (9, 2.44%). Most RTA victims were two-wheeler drivers/pillions, pedestrians, and victims of vehicle collisions. Overspeeding, reluctance to follow traffic rules, overcrowded roads using high beam lights, ditches or inappropriate bumps in roads, and lack of designated footpaths for pedestrians, particularly for urban dwellers, were traced to be major contributing factors for RTAs in different Indian studies [18,20,22-25], and the same may be attributed to the high incidence of RTA in our study. Assaults/interpersonal violence were the result of property-related conflict, relationship-related conflict, road rage, and interpersonal enmity. Blunt trauma, stab injury, and gunshot injury were the common types of injuries seen among assault victims. Falls included falls from heights as in industrial/construction work or from trees, slips in bathrooms and other places, and other agricultural activity-related falls. Domestic kitchen accidents, crop fires in rural areas, and fires resulting from electrical short-circuiting in appliances were among the leading causes of burns. Choking resulted from collapse of buildings, flyovers, and bridges as well as from suicidal or homicidal attempts. Other miscellaneous mechanisms of injury in our study were farm-related injury, falling objects, and animal bites, particularly dog bites. Our findings were in alignment with studies conducted by Menon et al. [20], Jain et al. [18], and Singh and Mishra [22], which also found RTA to be the most common mechanism of injury. However, the values from different Indian studies were variable, being 42.8% in Menon et al. [20], 74.20% in Jain et al. [18], and 67.3% in Singh and Mishra [22], attributable to variable study designs, sample sizes, and sources of data, as well as incomplete reporting of trauma. Casualty rate was highest in RTA (9.21%), followed by burns and choking, each accounting for about 7.69%.

Head injury was the most common type (132, 35.77%) of injury, followed by polytrauma (95, 25.74%). This finding was consistent with studies conducted by Kashid et al. [23] and Jain et al. [18] from India, who also reported head injury as the most common injury, seen in 40.5% and 50.80% of cases, respectively. Pathak et al. [24] and Mitra et al. [25] reported fractures, whereas Onyemaechi et al. [19] reported soft tissue injury as the most common type of injury. RTA, falls, assaults, and falling objects were the leading mechanisms of head injury. Head injury was seen more commonly among two-wheeler users who did not wear helmets; pedestrians; workers involved in industrial, construction, and mining activities; and assault victims. Polytrauma resulted from vehicle collisions, collapses of building/flyover/bridges, falls from heights, and assaults. Head injury led to traumatic brain injury, accounting for the highest case fatality rate (9.85%) in our study. The next highest casualties were seen in upper trunk injury (8.69%), followed by polytrauma (8.42%), abdomen and pelvis injury (4.76), upper limb injury (4.76%), and lower limb injury (3.57%). Two hundred twenty-one (59.89%) of the trauma victims had a hospital stay of <7 days and were associated with mortality of 4.52%, while 148 (40.11) of the victims had a hospital stay of ≥7 days and were associated with mortality of 12.16%.

Among the classes of drugs/products prescribed to trauma victims, antibiotics were the most common, and at least one antibiotic was prescribed to each patient. β-Lactams were the most commonly prescribed antibiotics, followed by aminoglycosides, metronidazole, clindamycin, azithromycin, and ciprofloxacin. Among β-lactams, ceftriaxone topped the list, followed by amoxicillin-clavulanate, cefotaxime, cefuroxime, cefixime, meropenem, and piperacillin-tazobactam. Most of the antibiotics were from the Access group; ceftriaxone, cefuroxime, cefixime, meropenem, azithromycin, and ciprofloxacin were from the Watch group, and none of them were from the Reserve group of WHO’s AWaRe classification of antibiotics.

Strength

This study attempted to generate data from our setting and geographical area on socio-demographic characteristics of trauma victims as well as various aspects of trauma. These data would serve as inputs for policymakers, healthcare providers, particularly primary care and family physicians, non-governmental organizations (NGOs), and even the general public, and help to take necessary actions to curb the incidents and improve the outcome. We also captured prescribing patterns of various classes of drugs/products, which, to the best of our knowledge, no study from our setting has attempted before.

Limitations

Being a single-center study, we cannot generalize our findings over a broader geographical area of eastern India. Since we could not capture data on pre-hospital trauma-related deaths and brought-in dead trauma victims, the mortality rate among trauma victims might be underestimated. The sampling technique used in this study was a non-probability sampling technique that might have introduced bias and affected the robustness of results. A substantial proportion of trauma patients was excluded due to incomplete records or leaving against medical advice, which might have introduced selection bias and might have affected the reported outcomes, including mortality outcomes.

Recommendations

The high burden of trauma and associated casualty in this study may be attributed to the lack of awareness among people about attributes of trauma, unsafe driving practices, overcrowded streets/highways, lack of strict rules on wearing protective gear at industrial/construction areas, lack of training about how to control fire in household and other situations and protect ourselves from it, and lack of training in people particularly elderly about how to protect ourselves from falling. Therefore, this study highlights the need for the government’s strategies to enhance awareness about the attributes of trauma and injury among the general public, to strengthen the trauma centers for better care and outcome, to develop equipped trauma centers with adequately trained medical personnel at district hospitals so that critical trauma victims can be treated locally and immediately, to work on street/road structure to free it from overcrowding and traffic mismanagement, to take stringent punitive actions on unsafe driving practice, to provide regular training on controlling fire, and to train elderly people to avoid falls. Equally important are recommendations for the public not to indulge in overspeeding, road rage, and unsafe driving practices as well as interpersonal enmity.

## Conclusions

In our study, the prevalence of trauma was 24%. Young men were the most affected. The most common time of incidents was morning and evening, and the most common place was streets/highways. Ambulance was the most common mode of transportation for trauma victims, most of whom arrived at the hospital between 1 and 20 hours after the incident. RTA was the most common mechanism of injury, whereas head injury was the most common type of injury. The overall mortality among trauma victims was about 8%. Antibiotics were the most consistently prescribed drugs and were either from the Access group or the Watch group, and none were from the Reserve group of WHO’s AWaRe classification.

## Appendices

**Table 5:** Data collection form

Patient identifier/CR no.: ____
Date and time of admission: __/__/____; __:__ DD/MM/YYYY; hr:min
Date and time of discharge: __/__/____; __:__ DD/MM/YYYY; hr:min
Study reference number: 362/IEC/IGIMS/2025
Study site: Department of Trauma and Emergency, IGIMS, Patna, India
Title of the study: Causes of admission, prescribing patterns, and outcomes among trauma patients visiting the Department of Trauma and Emergency at a tertiary care hospital in Bihar, India: a cross-sectional study
A. Socio-demographic data
Age: __ years
Sex:
Male ☐
Female ☐
Third gender ☐
Residence:
Rural ☐
Urban ☐
Education level:
Illiterate ☐
Primary ☐
Secondary ☐
Graduate ☐
Post-graduate ☐
Post-doctoral ☐
B. Vital clinical data
Blood pressure: __/__ mm Hg
Pulse rate: __ bpm
GCS score: __
C. Trauma-related data
Place of occurrence of incidents:
Street/highway ☐
Home ☐
Sports and athletics area ☐
Industrial/construction area ☐
School ☐
Farm ☐
Any other ☐ Please mention ____
Time of occurrence:
00:00-03:59 hours ☐
04:00-07:59 hours ☐
08:00-11:59 hours ☐
12:00-15:59 hours ☐
16:00-19:59 hours ☐
20:00-23:59 hours ☐
Unknown ☐
Mode of transportation:
Ambulance ☐
Autorickshaw/electric rickshaw ☐
Cabs/taxi ☐
Two-wheelers ☐
Police van ☐
Time to arrive at the trauma and emergency department:
Immediate (<1 hour) ☐
Within hours (1-24 hours) ☐
Within days (>24 hours) ☐
Mechanism of trauma:
Road traffic accident (RTA) ☐
Assaults/interpersonal violence ☐
Falls ☐
Burns ☐
Choking ☐
Any other ☐ Please mention ____
Type of injury:
Head ☐
Upper trunk ☐
Abdomen and pelvis ☐
Upper limb ☐
Lower limb ☐
Polytrauma ☐
Type of injury:
Head ☐
Upper trunk ☐
Abdomen and pelvis ☐
Upper limb ☐
Lower limb ☐
Polytrauma ☐
Duration of hospital stay:
<7 days ☐
≥7 days ☐
D. Drug/product prescribed
Name of drugs/products: ____
Generic ☐
Brand ☐
Route of administration:
Oral ☐
Intravenous (bolus or infusion) ☐
Intramuscular ☐
Subcutaneous ☐
Intradermal ☐
Any other ☐ Please mention ____
Dose and unit: ____
mg ☐
µg ☐
Any other unit ☐ Please mention ____
Frequency:
Once a day (24-hourly) ☐
Twice a day (12-hourly) ☐
Thrice a day (8-hourly) ☐
Four times a day (6-hourly) ☐
Any other ☐ Please mention ____
Duration of drug/product treatment: ____
Minutes ☐
Hours ☐
Days ☐
Months ☐
E. Outcome of trauma victims
Recovered ☐
Death ☐
If death, then date and time of death: __/__/____; __:__ DD/MM/YYYY; hr:min
Name of data collector ____
Signature of data collector ____
Date and time of completion of data collection: __/__/____; __:__ DD/MM/YYYY; hr:min
